# The stable oxygen isotope ratio of resin extractable phosphate derived from fresh cattle faeces†

**DOI:** 10.1002/rcm.8092

**Published:** 2018-04-11

**Authors:** Steven J. Granger, Yuguo Yang, Verena Pfahler, Chris Hodgson, Andrew C. Smith, Kate Le Cocq, Adrian L. Collins, Martin S. A. Blackwell, Nicholas J. K. Howden

**Affiliations:** ^1^ Rothamsted Research North Wyke Okehampton EX20 2SB UK; ^2^ Queen's School of Engineering University of Bristol Senate House, Tyndall Avenue Bristol BS8 1TH UK; ^3^ NERC Isotope Geoscience Laboratory British Geological Survey Keyworth Nottingham NG12 5GG UK

## Abstract

**Rationale:**

Phosphorus losses from agriculture pose an environmental threat to watercourses. A new approach using the stable oxygen isotope ratio of oxygen in phosphate (δ^18^O_PO4_ value) may help elucidate some phosphorus sources and cycling. Accurately determined and isotopically distinct source values are essential for this process. The δ^18^O_PO4_ values of animal wastes have, up to now, received little attention.

**Methods:**

Phosphate (PO_4_) was extracted from cattle faeces using anion resins and the contribution of microbial PO_4_ was assessed. The δ^18^O_PO4_ value of the extracted PO_4_ was measured by precipitating silver phosphate and subsequent analysis on a thermal conversion elemental analyser at 1400°C, with the resultant carbon monoxide being mixed with a helium carrier gas passed through a gas chromatography (GC) column into a mass spectrometer. Faecal water oxygen isotope ratios (δ^18^O_H2O_ values) were determined on a dual‐inlet mass spectrometer through a process of headspace carbon dioxide equilibration with water samples.

**Results:**

Microbiological results indicated that much of the extracted PO_4_ was not derived directly from the gut fauna lysed during the extraction of PO_4_ from the faeces. Assuming that the faecal δ^18^O_H2O_ values represented cattle body water, the predicted pyrophosphatase equilibrium δ^18^O_PO4_ (Eδ^18^O_PO4_) values ranged between +17.9 and +19.9‰, while using groundwater δ^18^O_H2O_ values gave a range of +13.1 to +14.0‰. The faecal δ^18^O_PO4_ values ranged between +13.2 and +15.3‰.

**Conclusions:**

The fresh faecal δ^18^O_PO4_ values were equivalent to those reported elsewhere for agricultural animal slurry. However, they were different from the Eδ^18^O_PO4_ value calculated from the faecal δ^18^O_H2O_ value. Our results indicate that slurry PO_4_ is, in the main, derived from animal faeces although an explanation for the observed value range could not be determined.

## 1 INTRODUCTION

Phosphorus (P) is an essential macro‐nutrient for plants and animals. It is fundamental to many biological processes because it is involved in energy transfer and is the constituent of several organic molecules.^1^ As such, it is essential to modern agricultural systems where it is applied both in the form of animal and plant wastes and as inorganic mineral fertilizers. However, in many parts of the world, a P surplus now exists such that more P is contained within the soil than is required by plants,^2^, ^3^ leading to increased P in soil water,^4^ and ultimately a proportion of this is lost to watercourses alongside any incidental losses that may occur from directly applied amendments.^5^ Even small increases of P in watercourses can have serious detrimental effects,^6^ causing eutrophication and eventually important shifts in ecosystems^7^, ^8^ and, for this reason, it is essential we understand better P chemistry, biochemistry and emissions from key sources in the landscape.

Stable isotope ratios have been used to track elements during transfers between different pools and to understand the respective roles of abiotic and biotic processes during these transfers.^9^, ^10^, ^11^ However, P has only one stable isotope and therefore the stable isotope ratio approach is not directly applicable. Despite this, a stable isotope approach has been developed which may shed more light on P cycling. This is because in the environment most P is bound to oxygen (O), forming anions such as orthophosphate (PO_4_^3−^), hydrogen phosphate (HPO_4_^2−^) and dihydrogen phosphate (H_2_PO_4_^−^) which can collectively be termed 'phosphate' (subsequently referred to as PO_4_ in the manuscript). This new approach uses the ratio between the ^18^O and ^16^O in PO_4_ (δ^18^O_PO4_ value) to understand better P sources and transformations. Comprehensive reviews have been written by Davis et al^12^ and Tamburini et al^13^ but, in short, at typical terrestrial temperatures and pH, and in the absence of biological activity, the P–O bonds in PO_4_ are stable. Therefore, bonds are only broken through biological mediation, and in these cases PO_4_ exchanges O with the ambient water within which it is in solution.^14^, ^15^, ^16^ The most important of these biological processes is generally considered to be that performed by pyrophosphatase, a ubiquitous intracellular enzyme that facilitates the hydrolysis of pyrophosphate. The hydrolysis of pyrophosphate leads to the formation of two PO_4_ ions incorporating one O atom from the ambient H_2_O. This process is extremely fast and leads to a complete O exchange between H_2_O and PO_4_ over time because PO_4_ as well as pyrophosphate can bind at the active site of pyrophosphatase.^13^ This enzyme‐catalyzed O exchange is subject to a thermodynamic isotopic fractionation, leading to a temperature‐dependent equilibrium value (Eδ^18^O_PO4_) which is predictable and initially described by Longinelli and Nuti^15^ but since refined by Chang and Blake^17^ and modified by Pistocchi et al:^18^

$$E\delta^{18}O_{\mathrm{PO}4}=-0.18T+26.3+\delta^{18}O_{H2O}$$

where Eδ^18^O_PO4_ is the stable O isotope ratio of PO_4_ at equilibrium in ‰, T is the temperature in degrees Celsius and δ^18^O_H2O_ is the stable oxygen isotope ratio of water in ‰.

For effective use of this approach for tracing the sources of PO_4_, the following criteria should be met:^12^

- The δ^18^O_PO4_ values for significant PO_4_ sources are well characterised (spatially and temporally)
- The individual sources of PO_4_ possess distinct δ^18^O_PO4_ signatures
- The δ^18^O_PO4_ values for PO_4_ sources are not equal to the Eδ^18^O_PO4_ values
- The δ^18^O_PO4_ signatures for PO_4_ sources are maintained and not rapidly transformed or modified by fractionation caused by metabolic processes.

One of the confounding issues surrounding this area of research is the narrow range of δ^18^O_PO4_ values that most PO_4_ sources have and that they often overlap or they are similar to the Eδ^18^O_PO4_ value.^13^, ^19^, ^20^ A recent study by Granger et al,^19^ which characterised different sources within a river catchment found that farm slurry, a mix of fresh and aged animal urine, faeces, bedding materials and other farm washings,^21^ had a relatively consistent δ^18^O_PO4_ value for water‐extractable PO_4_ despite its heterogenous composition. Furthermore, this study reported that its value was noticeably lower than the Eδ^18^O_PO4_ value in the rivers. Granger et al^19^ speculated that, given that the primary source of slurry PO_4_ was probably animal faeces, the δ^18^O_PO4_ value probably reflected the Eδ^18^O_PO4_ value of PO_4_ within the animal due to high microbial turnover, and that the Eδ^18^O_PO4_ value was strongly influenced by the higher body temperature relative to the ambient water temperature in the aquatic environment receiving the slurry.

In the present study, we sought to analyse fresh cattle faeces to establish its δ^18^O_PO4_ value, to see how consistent this value was, and whether it was similar both to the values of animal slurry already measured and to the calculated Eδ^18^O_PO4_ value for the animal. The forms of P in animal faeces can be split into three broad categories. Toor et al^22^ described many forms of P in animal faeces, although these can be more simply described as (i) organic P and (ii) inorganic P. However, their NaOH/EDTA extraction subsumes and incorporates a third form of P which is of interest when examining δ^18^O_PO4_ values – (iii) the microbial P. For the purposes of this study, we did not attempt to examine the δ^18^O_PO4_ values of organic forms of P, but, instead, aimed to characterise the inorganic 'free' PO_4_, and the 'microbial' PO_4_ of cattle faeces. There is no reported method for doing this in animal faeces so we attempted to apply and adapt an approach used for soils to test the following hypothesis: The δ^18^O_PO4_ value of inorganic 'free' PO_4_ and the 'microbial' PO_4_ will be the same and will reflect the Eδ^18^O_PO4_ value calculated for fresh cattle faeces.

## 2 EXPERIMENTAL

### 2.1 Sample collection

The details of the animals sampled are presented in Table 1. The animals sampled were being reared on the North Wyke Farm Platform^23^ and came from one of the three treatments which, individually, comprise a farmlet; (1) 'Legumes': sward improvement by reseeding with long‐term grass and white clover mixtures; (2) 'Planned reseeding': sward improvement through regular reseeding using new varieties of grass; and (3) 'Permanent pasture': sward improvement of the existing permanent grassland using artificial fertilisers (both other treatments are also fertilised). Samples were collected from seven animals whose ages ranged between 359 and 490 days old; six were male and one female, and five were Charolais crosses, one a Limousin cross, and one a Stabilizer.

**Table 1:** Information on the cattle from which faeces were sampled

Faeces ID	Animal ID	Date sampled	Gender	Breed	Age (days)	Farmlet
FP075/001	101621	27/6/17	Male	CHX	413	3
FP075/004	501569	28/6/17	Male	CHX	465	3
FP075/007	401561	29/6/17	Male	CHX	469	1
FP075/010	301623	3/7/17	Male	LIMX	417	2
FP075/013	601577	4/7/17	Male	ST	465	3
FP075/016	701536	5/7/17	Female	CHX	490	1
FP075/019	701634	6/7/17	Male	CHX	359	3

Breed codes: CHX = Charolais cross, LIMX = Limousin cross, ST = Stabilizer.

Farmlet codes: 1 = Legume enhanced, 2 = Planned reseeding, 3 = Permanent pasture.

Animals were not preselected for the study; simply, the first animal to defecate was selected. The animal ID number was noted and about 150 g of faeces was collected from the ground using sterile containers. Samples of fresh faeces were collected directly after being voided onto the soil surface in clean aluminium containers and returned immediately to the laboratory for sub‐sampling and preparation. First, a sub‐sample of 2–3 g faeces was placed into a 12‐mL glass exetainer, sealed and frozen at −20°C, ready for determination of its δ^18^O_H2O_ value. Secondly, a 1 g faeces sub‐sample for microbial analysis was placed in a 25‐mL polystyrene screw‐capped container (Sterilin, Newport, UK), diluted with 9 mL of Ringer's solution, (g L^−1^; sodium chloride, 2.25; potassium chloride, 0.105; calcium chloride 6H_2_O, 0.12; sodium bicarbonate, 0.05; pH 7.0; Oxoid, Basingstoke, UK), and stored at 4°C for analysis within 24 h. Thirdly, a 20–30 g sub‐sample was taken, placed in a pre‐weighed foil tray, weighed, and then dried to a constant weight at 105°C overnight to determine dry matter (DM) content.

### 2.2 Development of extraction methods for distinguishing inorganic and microbial PO_4_ in cattle faeces

The method development experiments for distinguishing inorganic and microbial PO_4_ were based on extraction methods described for soils;^24^, ^25^ whereby samples were extracted in a matrix of deionised water, or deionised water and hexanol, in the presence of anion‐exchange resins to collect 'free' PO_4_ and 'microbial' PO_4_, respectively. Tests using faeces found that there was no difference in the amounts of PO_4_ recovered from faeces with, or without, hexanol (results not presented). This suggested that either there was no microbiological content within the faeces, or that hexanol did not lyse the cells. As it seemed unlikely that there would be no faecal microbial content, it was hypothesised that osmotic stress was causing the lysis of most of the microbial cells present and therefore the addition of hexanol would not further increase the amount of extractable PO_4_. This hypothesis was based on the standard practice of microbiologists in using a buffered solution when extracting gut microbiology for culture.^26^, ^27^ Unlike soil microbiology, gut microbiology tends to be adversely affected in pure water and, to prevent this, the use of an isotonic diluent such as ¼ strength Ringer's solution is well established.

Ringer's solution contains mainly anions, to prevent the osmotic stress of the microbiology, so a recovery test was undertaken to see if it would adversely affect the ability of the anion resins to collect PO_4_. A PO_4_ spike was added to a container of Ringer's solution into which anion resins were placed. After a 16‐h shaking period, it was found that PO_4_ recovery was unaffected by the Ringer's solution (results not shown) and on this basis the study was continued.

#### 2.2.1 Microbiology

Determination of the number of bacteria was undertaken using the standard plate count method for Escherichia coli, a faecal indicator organism. The sample to be tested was diluted through serial dilutions to obtain a small number of colonies on each agar plate; 0.1 mL of the diluted sample was spread on the surface of a Membrane Lactose Glucuronide Agar (MLGA) (Oxoid) plate. Samples were initially vortex mixed before appropriate serial dilutions, from which 0.1 mL was spread plated aseptically. Once plates were dry, they were incubated at 44.0°C (±0.5°C) for between 18 and 24 h. After the total incubation period, all plates were examined and plates with between 30 and 300 colonies were counted.

### 2.3 Sample extraction

#### 2.3.1 Faecal PO_4_

Two further sub‐samples were extracted for PO_4_; (i) Resin PO_4_: 25–100 g placed in a 5‐L HDPE sealable bottle, diluted with 3 L Ringer's solution, and 72 anion‐exchange resin (VWR International Ltd, Lutterworth, UK) squares (4 cm × 4 cm) added; and (ii) Microbial PO_4_: 1–2 g placed in a 5‐L HDPE bottle and diluted with 3 L deionised water, and 72 anion‐exchange resin squares added. The bottles were placed on an orbital shaker set at 100 rpm, in a 4°C walk‐in refrigerator. After 16 h, the bottles were removed and the extracting solution sub‐sampled for microbial analysis by diluting 1 mL of extractant solution in 9 mL Ringer's solution and stored at 4°C before analysis within 24 h. Resins were then recovered by pouring the extraction solution from the 5‐L bottle though a 4 mm sieve ensuring that all resins were recovered from the bottle. As the sample was highly organic in nature we felt it necessary to test and, if needed, account for any potential hydrolysis of organic P during the extraction of PO_4_ from the resins. Resins from each extraction were divided into two sub‐sets of 36, placed in a 250‐mL polypropylene screw‐capped bottle and washed several times with their respective, fresh, matrix solutions. When clean, PO_4_ was liberated from the resins using 75 mL of 0.2 M nitric acid (HNO_3_). For each of the two sub‐sets of 36 resins collected from a single extraction matrix, δ^18^O_H2O_ unlabelled (−5.7‰) and labelled (+81.6‰) 0.2 M NHO_3_ was used to test for hydrolysis of organic P by the acid. The corrected δ^18^O_PO4_ value is then calculated using a revised version^18^ of the mass balance equation described by McLaughlin et al:^28^

$$\delta^{18}O_{\mathrm{PO}4}=\frac{\left(\delta^{18}O_{\mathrm{Psp}}*\delta^{18}O_{\mathrm{Aus}}\right)-\left(\delta^{18}O_{\mathrm{Pus}}*\delta^{18}O_{\mathrm{Asp}}\right)}{\left(\delta^{18}O_{\mathrm{Psp}}-\delta^{18}O_{\mathrm{Pus}}-\delta^{18}O_{\mathrm{Asp}}+\delta^{18}O_{\mathrm{Aus}}\right)}$$

where δ^18^O_PO4_ is the corrected final stable oxygen isotope ratio for PO_4_ considering the effect of any hydrolysis of organic P, δ^18^O_Psp_ is the stable oxygen isotope ratio of the PO_4_ collected using ^18^O‐spiked HNO_3_, δ^18^O_Pus_ is the stable oxygen isotope ratio of the PO_4_ collected using unspiked HNO_3_, δ^18^O_Aus_ is the stable oxygen isotope ratio of the water in the unspiked HNO_3_, and δ^18^O_Asp_ is the stable oxygen isotope ratio of water in the ^18^O‐spiked HNO_3_.

Phosphate in the extracts was converted into silver phosphate (Ag_3_PO_4_) using the purification protocol described by Tamburini et al.^29^ The process utilises a series of dissolution and precipitation reactions to isolate and purify dissolved PO_4_. The PO_4_ is precipitated first as ammonium phosphomolybdate before it is dissolved and reprecipitated as magnesium ammonium phosphate which is dissolved again. The resultant PO_4_ in solution is converted into Ag_3_PO_4_ through the addition of an Ag‐ammine solution which is then placed in an oven for 1 day at 50°C. Although the Tamburini protocol uses a DAX‐8 resin early in the extraction its use is not necessary unless organic contamination is present in the subsequent Ag_3_PO_4_ (F. Tamburini, personal communication).^30^

#### 2.3.2 Faecal water

Cryogenic extraction of faeces water was undertaken at the National Isotope Geosciences Laboratory, based at the British Geological Survey in Nottingham, UK. Frozen samples were placed in a U‐shaped vacuum tube (borosilicate glass), the sample containing side of which was immersed in liquid N_2_ to ensure complete freezing of sample water. The U‐tube was then evacuated to a pressure of <10^−2^ mbar, removing all the residual atmosphere. Once under stable vacuum, the U‐tube was sealed, removed from the vacuum line and the sample side of the tube placed in a furnace at 100°C. Sample water collection was achieved by immersing the opposite side of the glass U‐tube in liquid nitrogen, forcing evaporated sample water to condense and collect. This setup was maintained for at least 1 h to ensure complete water transfer. Sample water was collected and stored refrigerated in 1.5‐mL vials with no headspace until isotope analysis. Samples were weighed before and after extraction to assess whether they had been successfully dried.

### 2.4 Sample analysis

#### 2.4.1 Phosphate

Phosphate concentrations were determined colourimetrically on an Aquachem 250 analyser (Thermo Fisher Scientific, Waltham, MA, USA) using a molybdenum blue reaction^31^ after they had been diluted (typically 1/10^th^) to avoid any acid interference with the molybdenum chemistry.

#### 2.4.2 Isotopes

Measurement of the PO_4_^18^O/^16^O ratio was undertaken by weighing approximately 300 μg of Ag_3_PO_4_ into a silver capsule to which a small amount of fine glassy carbon powder was added.^29^ The sample was converted into carbon monoxide by dropping it into a thermal conversion elemental analyser (ThermoFinnigan, Bremen, Germany) at 1400°C; the resultant carbon monoxide mixed with a helium carrier gas passed through a GC column into a Delta + XL mass spectrometer (ThermoFinnigan). The δ^18^O_PO4_ values were calculated by comparison with an internal Ag_3_PO_4_ laboratory standard, ALFA‐1 (ALFA‐1 = δ^18^O VSMOW value of +14.2‰). In the absence of an international Ag_3_PO_4_ reference material, we derived this value for ALFA‐1 by comparison with the Ag_3_PO_4_ standard 'B2207' (Elemental Microanalysis Ltd, Okehampton, UK), which has been measured in an inter‐laboratory comparison study to have a δ^18^O value of +21.7‰ versus VSMOW. Samples were run in triplicate, with a typical precision σ ≤0.3‰. Sample purity was assessed by determining the CO yield compared with the yield of Ag_3_PO_4_ standards, and samples were rejected where this differed by 10%.

Faeces water δ^18^O_H2O_ values were determined on an Isoprime Aquaprep coupled to an Isoprime 100 dual‐inlet isotope ratio mass spectrometer (Isoprime Ltd, Cheadle Hulme, UK) through a process of headspace CO_2_ equilibration with water samples. The isotope ratios are reported as δ^18^O_H2O_ values versus VSMOW, based on comparison with laboratory standards calibrated against IAEA standards VSMOW and SLAP, with analytical precision typically σ ≤0.05‰.

### 2.5 Statistical analysis

All statistical analyses were conducted in R.^32^

## 3 RESULTS

### 3.1 Faecal properties

The fresh faeces were found to have a DM ranging from 9.3 to 16.6% with a mean of 11.4% (±2.5) while the δ^18^O_H2O_ values ranged between −1.19 and +0.41‰ with a mean of −0.73‰ (±0.65) (Table 2). The amounts of PO_4_ collected from faeces when using Ringer's solution ranged from 67 to 93 μg PO_4_‐P g^−1^ DM with a mean of 78 (±9.1) μg PO_4_‐P g^−1^ DM. This was found to be significantly less (t_6_ = −8.03; p <0.001) than that collected using deionised water which ranged from 3885 to 8635 μg PO_4_‐P g^−1^ DM with a mean of 5713 (±1856) μg PO_4_‐P g^−1^ DM.

**Table 2:** Properties of the different fresh faeces samples collected

Faeces ID	Fresh faeces		Ringer's solution			Deionised water		
	%DM	δ^18^O_H2O_ values (‰)	Faeces used (g)	μg PO_4_‐P recovered	μg PO_4_‐P g^−1^ DM	Faeces used (g)	μg PO_4_‐P recovered	μg PO_4_‐P g^−1^ DM
FP075/001	16.6	‐	23.4	259	67	2.2	3145	8635
FP075/004	10.0	‐	28.8	247	86	1.8	699	3885
FP075/007	9.3	−1.19	23.5	204	93	1.6	772	5161
FP075/010	12.6	−0.85	99.1	874	70	1.7	1431	6686
FP075/013	10.0	−1.02	100.2	805	80	2.0	840	4181
FP075/016	10.6	−0.98	100.4	786	74	1.7	739	4109
FP075/019	10.8	0.41	100.2	814	75	1.5	1192	7331

### 3.2 Faecal microbiological content

Fresh cattle faeces had E. coli concentrations ranging from 6.1 to 7.85 CFU g^−1^ DM (Table 3). The concentrations of E. coli in the two extracting solutions ranged from 5.73 to 7.71 CFU g^−1^ DM in Ringer's solution and from 5.85 to 8.02 CFU g^−1^ DM in deionised water. There was no significant difference in E. coli concentrations between raw faeces, Ringer's solution and deionised water.

**Table 3:** Colony‐forming units (CFU) for E. coli in raw faeces, a Ringer's solution extraction and a deionised water extraction expressed in per g of faecal dry matter (DM)

	Ringers solution		
Faeces ID	Raw faeces	log_10_ CFU g^−1^ DM	Deionised water
FP075/001	6.28	6.38	6.22
FP075/004	7.85	7.71	8.02
FP075/007	7.01	6.99	7.05
FP075/010	6.10	5.73	5.85
FP075/013	7.10	7.22	7.04
FP075/016	6.93	7.08	7.46
FP075/019	7.38	7.35	7.63

### 3.3 Extractable faecal δ^18^O_PO4_ values

To assess whether organic P had been hydrolysed by the 0.2 M HNO_3_ resin elution solution, the δ^18^O_PO4_ values obtained following extraction with ^18^O‐labelled and unlabelled HNO_3_ were analysed statistically and it was found that no significant difference occurred between labelled and unlabelled acid elution for extractions with either Ringer's solution (t_3.358_ = −1.2012; p >0.05) or deionised water (t_11.606_ = 0.6995; p >0.05). It was concluded therefore that there was no need to correct data using the equation described by McLaughlin et al.^28^ Instead, a mean of the spiked and unspiked values was used to report the resin‐extractable δ^18^O_PO4_ values. The δ^18^O_PO4_ values for the PO_4_ extracted from faeces are presented in Table 4. The δ^18^O_PO4_ values for PO_4_ extracted using Ringer's solution for the first three samples are not presented as the amount of some of them was too small for standard Ag_3_PO_4_ precipitation. Of the remaining four faecal samples the values ranged from +12.0 to +19.8‰ with mean values between +12.1 and +16.3‰. The values for the seven samples extracted in deionised water ranged from +12.9 to +15.6‰ with mean values of +13.2 and +15.3‰. The greatest variation between labelled and unlabelled acid δ^18^O_PO4_ elution values occurred in the Ringer's solution dataset with the mean difference of the labelled acid extraction being +2.1‰. This result, however, was strongly influenced by one anomalously high labelled acid δ^18^O_PO4_ value of +19.8‰, leading to a difference of +6.9‰. This sample also had a slightly higher oxygen yield indicating that it was not pure Ag_3_PO_4_ which could explain the relatively high difference between the δ^18^O_PO4_ values of labelled and unlabelled acid extraction. The differences observed in the deionised water labelled and unlabelled acid elution were far smaller and ranged between −1.8 and +1.4‰ with a mean of −0.3‰. Statistical analysis of the two sets of paired data shows that there was no difference between the δ^18^O_PO4_ values obtained following extraction using Ringer's solution and that using deionised water (t_3.463_ = 0.0785; p >0.05).

**Table 4:** Measured and mean δ^18^O_PO4_ values of PO_4_ collected from seven fresh cattle faeces samples using anion resins in either Ringer's solution or deionised water

	Ringer's solution			Deionised water		
	Unspiked	Spiked	Mean	Unspiked	Spiked	Mean
Faeces ID	δ^18^O_PO4_ (‰)					
FP075/001	‐	‐	‐	+15.6	+15.0	+15.3
FP075/004	‐	‐	‐	+12.9	+13.4	+13.2
FP075/007	‐	‐	‐	+15.3	+13.5	+14.4
FP075/010	+13.5	+13.4	+13.4	+14.2	+14.2	+14.2
FP075/013	+12.3	+12.0	+12.1	+13.7	+13.5	+13.6
FP075/016	+12.9	+19.8	+16.3	+13.9	+15.3	+14.6
FP075/019	+14.3	+16.3	+15.3	+15.1	+13.3	+14.2

## 4 DISCUSSION

### 4.1 Microbiological content

The concentrations of E. coli reported here are consistent with those reported in the literature for beef cattle faeces.^33^, ^34^, ^35^ The use of ¼ strength sterile Ringer's solution before bacteriological examination is well established^26^, ^27^ to effectively protect bacterial cells from the osmotic shock that they would experience when being suspended in sterile water. However, the new data from this study (Table 3) indicate that there was no difference between Ringer's solution and deionised water and that the microbial cells were thus not lysed in water and that the extracted PO_4_ in both cases does not represent 'microbial' PO_4_ released through cellular breakdown during the extraction process but, instead, 'free' PO_4_.

### 4.2 Resin‐extractable PO_4_

The amounts of PO_4_ extracted in deionised water were significantly higher than in Ringer's solution. This finding is at odds with the initial recovery test undertaken on PO_4_ in a pure Ringer's solution matrix. However, it would seem that the combination of organic material, faecal anions, and the anions within the solution itself significantly reduced the recovery of PO_4_ on the resins in a way that did not occur in just the Ringer's solution alone. This interference raises questions about the validity of the δ^18^O_PO4_ values of PO_4_ recovered in this solution due to potential unknown fractionations that might occur as a result of preferential adsorption/desorption of the lighter/heavier isotopologues.^36^ The microbiological analysis showed that cell lysis and rupture did not occur in either extraction (Table 3). Therefore, the results derived from the Ringer's solution extraction are not considered further in this discussion, as it apparent that the method for distinguishing microbial PO_4_ from inorganic PO_4_ (as defined earlier) requires further development.

### 4.3 Faecal water

The fresh faeces %DM values are consistent with those reported elsewhere for cattle grazing pasture.^37^ The cattle's main source of water is via drinking troughs supplied using ground water originating from a local borehole. The δ^18^O_H2O_ value of the groundwater is relatively stable and will represent an integrated value of the annual precipitation supplying it. At this location, the δ^18^O_H2O_ value is predicted to be between −5.5 and −6.0‰.^38^ The drinking troughs are refilled with fresh water every time that an animal drinks from them and therefore we do not consider deviations from the groundwater δ^18^O_H2O_ value due to evaporative losses as important. Abeni et al^39^ also found that summer and winter drinking water δ^18^O_H2O_ values did not differ greatly despite the increased temperatures. Water is also ingested as metabolic water in food, which is likely to be isotopically heavier than local meteoric water due to fractionation;^40^ however, the main source of water for the animal is considered to be that supplied by the drinking troughs. Abeni et al^39^ showed that the δ^18^O_H2O_ values of various forms of body water in cattle were from 4.2 to 7.9‰ heavier than in drinking water in the summer and that for faecal water they were from 4.8 to 7.7‰ heavier. The measured δ^18^O_H2O_ value in faeces in this study was found to be up to 6.4‰ heavier than in groundwater and this was not unexpected as demonstrated by the model proposed by Bryant and Froelich.^40^ Water lost via breath water vapour and transcutaneous water vapour will be isotopically fractionated, leading to an increase in body water δ^18^O_H2O_ values while water lost via pathways such as urine, faeces and sweat will be similar and thus have similar δ^18^O_H2O_ values to that of the animal's body water. The increase in δ^18^O_H2O_ value will also be more pronounced in the summer when temperatures are higher.^39^

### 4.4 Theoretical animal Eδ^18^O_PO4_ values

The use of Eδ^18^O_PO4_ values is widespread within the δ^18^O_PO4_ community to benchmark measured values with values that have potentially lost their original signal through intracellular cycling, specifically through the enzyme pyrophosphatase. However, there is much uncertainty as to how relevant this theoretical equilibrium is in many situations, and we acknowledge that in terms of animal gut processes other cycling pathways may predominate.

**Figure 1:**
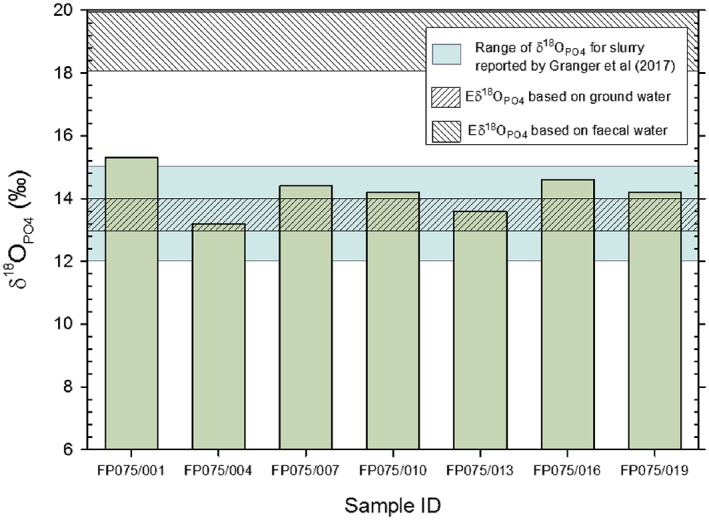
The range of δ^18^O_PO4_ values for deionised water extracted fresh faeces compared with (i) the reported values for agricultural slurry, (ii) the Eδ^18^O_PO4_ for cattle assuming body water δ^18^O_H2O_ is equivalent to ground water and, (iii) the Eδ^18^O_PO4_ for cattle assuming body water δ^18^O_H2O_ is equivalent to faecal water [Color figure can be viewed at http://wileyonlinelibrary.com]

The normal temperature of cattle is 38.6°C, with anything outside a range of 38.0 to 39.2°C indicating ill health.^41^ When combined with the range of δ^18^O_H2O_ values measured in faeces and with the range expected for the ground/drinking water in the region, a Eδ^18^O_PO4_ range of values from +13.2 to +14.0‰ is expected, assuming that the body water δ^18^O_H2O_ value is similar to that of ground water and +18.1 to +19.9‰ if the δ^18^O_H2O_ values within faeces are used and are taken to represent the animal body water (Figure 1).

### 4.5 Extractable faecal δ^18^O_PO4_ values

As it was shown that the resin‐extractable PO_4_ was not derived directly from the lysis of microbial cells, it was not possible to compare 'free' PO_4_ with 'microbial' PO_4_. However, the δ^18^O_PO4_ values of the 'free' PO_4_ ranged between +13.2 and +15.3‰ which are very similar to those reported for slurry PO_4_ by Granger et al^19^ which ranged between +12.0 and +15.0‰ despite being extracted differently and representing a much more heterogeneous source material (Figure 1). There was no apparent relationship between the δ^18^O_PO4_ values and the animal variables; however, the scope of the study was too limited to investigate variables such as age, gender, breed, etc. The δ^18^O_PO4_ values reported within this study indicate that the slurry δ^18^O_PO4_ values are caused by the PO_4_ in animal faeces. The δ^18^O_PO4_ values of the faeces themselves, however, are at or slightly above the range of Eδ^18^O_PO4_ values based on the ground/drinking water δ^18^O_H2O_ values. However, all the values are at least 2.8‰ lower that the Eδ^18^O_PO4_ value range calculated from the δ^18^O_H2O_ value of faecal water, water that should be far more representative of the body water of the animal.^40^ It is unclear why this is the case without further work being carried out to investigate animal P food sources and metabolic processes within the animal.

## 5 CONCLUSIONS

- The extractable PO_4_ from fresh cattle faeces was lower using Ringer's solution than deionised water. However, this did *not* appear to be because of microbial cellular lysis in the deionised water extraction. It would appear to be due to some form of interference between the Ringer's solution ions, compounds in the faeces and the anion resin sheets. Because of this it was *not* possible to differentiate 'microbial' PO_4_ and 'free' PO_4_, and their respective δ^18^O_PO4_ values. As it has been shown that deionised water does not lyse the microbial cells it would be worth repeating the study using the more traditional resin PO_4_ extraction in a water/hexanol extraction solution to extract 'microbial' PO_4_ and to also use the microbial assays described to establish if this occurs.
- The δ^18^O_PO4_ values of fresh cattle faeces, under the conditions reported in this study, ranged between +13.2 and +15.3‰ which are consistent with those reported elsewhere for agricultural animal slurry.
- The δ^18^O_PO4_ values are similar to the Eδ^18^O_PO4_ value calculated for within the animal using the δ^18^O_H2O_ value of groundwater. However, they are at least 2.8‰ lower than the Eδ^18^O_PO4_ value range calculated using faecal water as a proxy for the animals' body water.
- There were no apparent relationships between the animal variables and the δ^18^O_PO4_ value. However, to examine these, a more detailed study is required which should also include other animals for which few data exist in the literature.
